# Deriving Public Innovation Capacity: Evidence From the Korean Public Sector

**DOI:** 10.3389/fpsyg.2022.898399

**Published:** 2022-06-02

**Authors:** Min Young Kim, Sang Woo Kim

**Affiliations:** ^1^Jeonju University, Jeonju, South Korea; ^2^Sangmyung University, Seoul, South Korea

**Keywords:** public innovation capacity, Korean public sector, Delphi analysis, individual, middle manager, organizational levels

## Abstract

By actively coping with changes, a government providing public services can also improve the quality of those public services and help citizens improve their quality of life in the face of rapidly changing social structures, environments, and values. Accordingly, this study will typologize public innovation capacity (PIC) in terms of the individual, middle manager, and organizational levels. This study typologizes public innovation capacity in terms of the individual, middle manager, and organizational levels through mini-round Delphi analysis and exploratory factor analysis and confirmatory factor analysis using survey. This capacity is a precondition of the specificity of the public sector (generating public interest by providing public services) and universal value of HR (human resource) research (creating performance). It provides the basic capacity within the public sector to enhance the quality of public services and create positive outcomes.

## Introduction

By actively coping with changes, a government providing public services can also improve the quality of those public services and help citizens improve their quality of life in the face of rapidly changing social structures, environments, and values (Gieske et al., 2019). Osborne and Plastrik (1998) defined innovation in the public sector as the fundamental transformation of public systems and organizations to create dramatic increases in their effectiveness, efficiency, and capacity to innovate and stressed its importance. Walker (2007) argued that the public sector pursues innovation based on laws and systems or does not have an established concept of innovation, which is why it is difficult for it to seek innovation in a groundbreaking way. The external environment, supply-demand principle, profit generation, and regulations of the public sector are different from the private sector’s circumstances; thus, innovation might not be considered a priority in the public sector (Lewis et al., 2018). There are various reasons to seek innovation in the public sector, but the key is to provide high-quality public services in the right place and at the right time (Demircioglu and Audretsch, 2019; Wynen et al., 2019), which could create public value and pursue the public interest.

The most important factor in change management that induces organizational innovation is the recognizing the need for change among members in response to a sense of crisis that if they do not change they will be eliminated (Hayes, 2022). However, unlike private organizations, public organizations are relatively less sensitive to changes in the environment (Longo and Cristofoli, 2008). In particular, in Korea, since the stability of public organizations is guaranteed according to the law, it is relatively difficult to create a sense of crisis that organizations may disappear or become unemployed, even if they do not change compared to the private sector (Kim, 2012).

After the New Public Management (NPM) was implemented, the concept of innovation was introduced to the public sector (De Vries et al., 2018), but there have been difficulties in producing and spreading innovation in the public sector due to its low compensation and high risk (Bugge and Bloch, 2016). Moreover, to solve the chronic problems connoted by public services such as legal limitations on the target and scope of public services and a decrease in publicness for contracting-out and to maximize the effectiveness of public services by efficiently using internal resources that have already been obtained, it is necessary to actively establish strategies to seek innovation (Demircioglu and Audretsch, 2019; Meijer, 2019).

Over the last decade we have meet massive dynamics in key organization circumstances such as a 4th industrial revolution, big data, and so on. However, due to the lack of innovators and discussions about their roles, such efforts could not directly lead to the enhanced quality of public services (Hjelmar, 2019). Public organizations have led innovation in managerial aspects such as implementing new management techniques or establishing innovation agencies rather than pursuing fundamental innovation of the actors in organizations; that is why there have been limitations in internalizing innovation and producing results thereof. Here, we try to highlights how innovation can build bridges across organizations to response organizational environment change. In addition, in the case of Korea, it is different from other countries in that public officials were the driving force behind the country’s innovative change (Kim, 2012). However, while the old generation led the nation’s development through dedication to the organization and continuous innovation efforts, the new generation pays more attention to values such as personal life satisfaction rather than organization commitment or innovation (Park and Park, 2018). In doing so, this study judges that the innovation capability of public organization’s members is most important for the sustainable development of Korean society.

**FIGURE 1:**
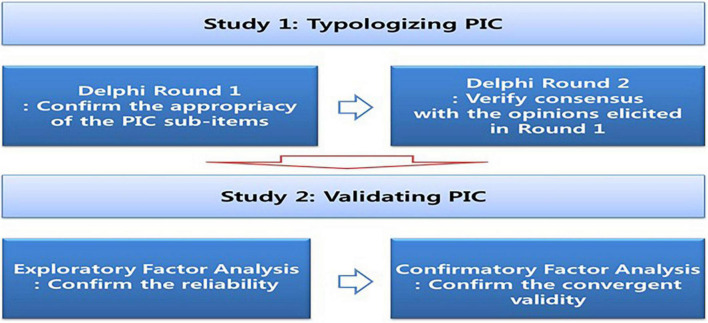
Research process.

Previous studies have actively discussed various factors that affect the innovative behaviors of organizational members from both theoretical and practical viewpoints, rather than focusing on innovation capacity (e.g., De Vries et al., 2018; Cinar et al., 2019; Wynen et al., 2019). In particular, the study focused on the difference between public innovation from the NPM perspective and cooperative innovation based on governance (Sørensen and Torfing, 2011; Sørensen, 2012). More specifically, it has been empirically proved that independence of wages and duties as well as procedural legitimacy have a positive effect on the innovative behaviors of members (Ramamoorthy et al., 2005). Holzer and Callahan (1998) proved that support from top managers, work-related training, organizational structure, a system to promote innovation, the resources used in the organization (human and material), compensation, and incentives had effects on innovative behaviors; in addition, organizational learning and an appropriate level of job stress also turned out to have an effect on innovation (Lemon and Sahota, 2004). Specifically, it focuses on the positive effects on organizations equipped with innovation capabilities rather than the components of innovation capabilities that individuals should possess. Therefore, this study can be differentiated from previous studies in that it focuses on the innovation capability itself. Accordingly, this study will typologize public innovation capacity (PIC) in terms of the individual, middle manager, and organizational levels (see Figure 1). This capacity is a precondition of the specificity of the public sector (working in public interest by providing public services) and universal value of HR research (creating performance). It provides to secure the basic capacity within the public sector in order to enhance the quality of public services and create positive outcomes.

This research consists of five parts. First, the introduction briefly overviews the importance of innovation in the public sector; why the public sector needs to verify the PIC. The second section of literature review draws the institutional and theoretical backgrounds of PIC and the concepts of PIC. Third, the study applies the Delphi method for the purpose of gathering the viewpoints of experts in the field of PIC. Fourth, to determine the factor structure of PIC at the individual, middle manager, and organizational levels, this study tests EFA (exploratory factor analysis) and CFA (confirmatory factor analysis). Finally, the discussion part suggests research implications and limitations in the HR (human resource) field.

## Literature Review

### Institutional and Theoretical Background of Public Innovation Capacity

As the roles of the public sector increased and terms such as “welfare” or “administrative state” became generalized and widely used, issues began to arise due to the public sector’s intervention in various parts of society. Moreover, the negative roles and actions of the public sector were expressed in the form of citizens’ distrust, conflicts, or economic crises. Accordingly, there have been discussions about public innovation based on the hostility toward the public sector’s huge role and awareness of the need to maximize its performance and efficiency (Sørensen and Torfing, 2011; Cannaerts et al., 2016; Demircioglu and Audretsch, 2019). Innovation efforts in the public sector based on NPM perceived the private sector as a competitor, implemented private management techniques in the public sector, and strived to create a competitive environment (Bloch and Bugge, 2013; Demircioglu, 2017; Demircioglu and Audretsch, 2019). As such, NPM is an important element in designing the direction of innovation in the public sector and has encouraged the development of various types of innovation (De Vries et al., 2018).

The PIC mainly consists of the role and functional shift of the government, entrepreneurial-customer-centric e-government, and privatization; it also mainly consists of efficiency, democracy, transparency, decentralization, and reform of organizational structures and management skills (Walsh et al., 2016; Demircioglu, 2017). After the faith in the efficiency of bureaucracy was shattered, NPM was implemented with a focus on improving efficiency in the public sector by adopting business logic and management skills. This raised fundamental questions about the efficiency of the public sector and contributed to encouraging government innovation or reform. In particular, innovation in terms of NPM, characterized by its emphasis on performance and accountability through performance management, downsizing, and expansion of privatization, can be considered as a movement that breaks away from the conventional government-led administration (Andersen and Jakobsen, 2018).

As NPM received attention as a typical model of public innovation capacity, it positioned itself as a new management strategy in the public sector. Meanwhile, countries such as the United Kingdom institutionalized the participation of stakeholders, including citizens, in decision making and emphasized government innovation for prompt government measures, whereas the United States maintained a certain level of the key parts of NPM while emphasizing New Public Service, which focuses on humanism, serving citizens, and communitarianism (Denhardt and Denhardt, 2000). Park and Joaquin (2012) discovered that the reform values of NPM (effectiveness) and post-NPM (humanistic) coexist in public institutions in the United States. This indicates that NPM reform values and post-NPM reform values are not replaced by a single value but are mediated according to the government’s policy director, or values are pursued like the ebb and flow.

Currently, the innovation value of NPM still has a strong influence in discussions of innovation in the public sector of Korea, but it is necessary to utilize innovation with more diverse management techniques in order to more sensitively meet the diversified needs and demands. This indicates that a one-size-fits-all model or value can no longer meet all needs in the public sector (Park and Joaquin, 2012). Accordingly, this study will also discuss PIC in terms of the institutional backgrounds of NPM and post-NPM.

### Public Innovation Capacity

Previous studies discussed the concept of innovation capacity as human skills demonstrating technical proficiency (Lall, 1992); resources to discover a new environment (Szeto, 2000); future-oriented developmental competency; the ability to create new resources; human, material, and environmental capabilities to achieve outcomes; and the capacity to create new resources by integrating organizational resources (Pierre and Fernandez, 2018). To summarize such definitions, innovation capacity includes the expertise of members and organizational resources that can create new resources in a future-oriented view.

However, while the literature on innovation capacity in the private sector has a long history, consideration of public innovation only began in the 1980s (Walker, 2007). To make public innovation work, governments require a PIC (Meijer, 2019, p. 618). Also, to promote the quality of life and satisfaction of citizens, a public sector that demonstrates innovation (Wynen et al., 2019) must reinterpret new resource creation in terms of creating public interest and spreading social values. The concepts of PIC are based on theories of public innovation (the significance of individuals, organizations, and networks in public innovation) and innovative systems (the role of government systems in public innovation) (Meijer, 2019, p. 617).

This study classifies PIC into three levels—individual, middle manager, and organizational—in terms of the internal resources or capital in an organization. Moreover, this study presumed that the three levels of PIC may interact with one another and increase the performance of public service. Individual PIC that can be converted into human capital is defined as the capacity of organizational members to secure task expertise and perform their tasks creatively (Snell and Dean, 1992). Middle managers’ PIC can be understood as the social capital of the organization, defined as the capacity of middle managers to motivate their junior staff by interacting with them and inducing cooperation within the organization (Youndt and Snell, 2004). Finally, PIC at the organizational level is defined as the organizational structure or culture that uses human and material resources in the right place at the right time, strategically manages human resources, and actively deals with changes in the external environment in terms of organizational capital (Pierre and Fernandez, 2018). In sum, this study defines PCI as the “task expertise of organizational members in providing high-quality services for citizens and the role of middle managers in displaying, maintaining, and managing this expertise and organizational resources.”

In this study, the reason for classifying PIC into three dimensions, such as individuals, middle managers, and organizations, that is, we assumed the sub-attributes of innovation capacity will be different depending on the entity expresses it. The research was conducted that assuming the PIC at the individual, middle manager, and organizational level as the organization capital. Specifically, PIC at the individual level that can be replaced by human capital is defined as organizational members securing work expertise and performing work creatively (Snell and Dean, 1992). Middle manager’s PIC can be understood as the social capital of an organization, which is defined as the ability of middle managers to motivate and induce collaboration within the organization through interactions with subordinates (Youndt and Snell, 2004). Organizational PIC is interpreted in terms of organizational capital. In other words, it is defined as an organizational structure and culture that utilizes human and material resources in the right place, strategically manages human resources, and actively responds to external environmental changes. In doing so, we attempt the following research question:

*Research Question: Is public innovation capacity composed of sub-dimensional capacity constructs, i.e., individual, middle manager, and organizational levels? Are these latent variables statistically and empirically distinct?*

## Study 1: Typologizing Public Innovation Capacity

### Research Method

#### Participants

This study applied the Delphi method^1^ for the purpose of gathering the viewpoints of experts^2^ in the field of PIC. The principles of this method are based on the notion of collective wisdom in decision making, assuming that the combined opinions of several people come closer to the truth than the opinion of one individual (Fuermaier et al., 2019, p. 341). For the Delphi panel, this study selected a total of 14 participants, consisting of 4 public officials who are in charge of human resource departments in public organizations, 2 public officials working in capacity building training at human resource development institutes, and 8 academics who specialize in organizational and personnel management (see Table 1).

**TABLE 1:** Characteristics of the Delphi participants.

Category	No.	Gender	Age	Job status	Job tenure	Education
Academic expert	1	Male	40s	Professor	Over 10 years	Doctor
	2	Male	40s	Associate Professor	Over 5 years	Doctor
	3	Female	30s	Assistant Professor	Over 5 years	Doctor
	4	Female	30s	Senior Researcher	Under 3 years	Master
	5	Female	30s	Senior Researcher	Under 3 years	Master
	6	Female	30s	Senior Researcher	Over 5 years	Doctor
	7	Male	30s	Senior Researcher	Under 3 years	Master
	8	Male	40s	Senior Researcher	Over 5 years	Doctor
Public officials who are in charge of human resource departments	9	Male	40s	Grade 6	Over 10 years	Bachelor
	10	Male	40s	Grade 4	Over 10 years	Bachelor
	11	Male	40s	Grade 5	Over 15 years	Bachelor
	12	Male	30s	Grade 7	Over 3 years	Bachelor
Public officials who are working at human resource development institutes	13	Male	50s	Grade 4	Over 15 years	Master
	14	Female	40s	Grade 7	Over 10 years	Bachelor

#### Delphi Process

According to Kim (2015), the Delphi method is carried out in four rounds, but recently, the “mini-round Delphi,” which has only two rounds, has frequently been used to compensate for the Delphi method’s weakness of requiring too much money and time. Therefore, in this study, I employed a two-round modified Delphi method to gather the views of the expert panel in terms of PIC.

The first-round Delphi (April 4–16, 2018)^3^ was the round in which PIC was assessed based on the experience and knowledge of the experts. The questionnaires were developed based on the literature review on innovation capacity in Korean private or public organizations in Round 1. To confirm the appropriacy of the PIC sub-items, I designed semi-open questions with options like agree, eliminate, and modify (if modifying, I asked for a comment) to give the experts the freedom to present their views and contribute new concepts. Individual characteristics were collected in Round 1.

The second-round Delphi (April 23–30, 2018) pursued the goal of verifying the consensus on the opinions elicited in Round 1. I asked the experts to state their agreement with the issues proposed in Round 2 using a five-point Likert scale (ranging from strongly disagree to strongly agree).^4^ All rounxds were sent to each expert via e-mail to combine the PIC sub-items.

### Research Results

#### Results of Delphi Round 1

If the revised opinions were deemed appropriate after the researcher examined the opinions of the expert panel, these opinions were actively reflected in revising the sub-factors of individual PIC. To begin with, individual attribute capacity was renamed “individual characteristic capacity.” Items with redundant meanings such as ethics and accountability as public officials in the sub-indexes of individual characteristics were revised into the single item of responsibility (administrative, legal, professional, and political). Moreover, indexes that connoted the attributes of different capacities such as customer/beneficiary-oriented and business minded were eliminated from individual attribute capacity. Thus, the sub-indexes of individual attribute capacity were ultimately reduced from 12 to 9. Job performance capacity consisted of a total of 12 sub-indexes after adding displays of self-leadership, policy, and business briefing skills. It was pointed out that the organizational management capacity group was inappropriate as a sub-group of individual innovation capacity, and thus it was eliminated and the sub-indexes such as mediation and integration skills or exertion of influence over stakeholders were absorbed by interpersonal capacity. Finally, interpersonal capacity was renamed as “relationship building capacity,” and the sub-indexes absorbed from the organizational management capacity group were added, ultimately forming seven items in total.

The individual attribute capacity of middle managers was renamed “individual characteristic capacity.” Items with redundant meanings such as ethics and accountability as public officials in the sub-indexes of middle manager characteristics were revised into the single item of responsibility (administrative, legal, professional, and political). Moreover, items such as persuasive power and initiative were eliminated, and new sub-indexes such as emotional intelligence or positive psychological capital (resilience, hope, self-efficacy, and optimism) were created. Thus, the sub-indexes of middle manager attribute capacity were ultimately increased from 9 to 10. Items that were included in organizational management capacity such as coaching/feedback and motivation were added to job performance capacity, as well as new sub-indexes such as policy development and ability to predict the future. Thus, the sub-indexes ultimately increased from 7 to 10. As for organizational management capacity, sub-indexes that moved to job performance capacity such as coaching/feedback and motivation and new items such as agile responses to change and strategic human resource management (talent hunt, development, and use) were added, and thus the sub-indexes decreased from 12 to 9. Finally, interpersonal capacity was renamed “relationship building capacity,” and items such as interpersonal relations and teamwork were eliminated, while items such as participation in official and unofficial mentoring, attentive listening of members’ opinions, and followership were added. Thus, the sub-indexes ultimately increased from four to five.

For the sub-indexes of organizational goal capacity, sharing and achievement of vision was revised to collection and sharing of vision and design of the direction for organizational goals was revised to development of effective policies to achieve organizational goals. There was a total of five sub-indexes, which was the same as before. Job performance capacity was renamed “individual characteristics and job performance capacity of members,” and items such as ownership, display of self-leadership, and will to fulfill social values were added. Items with similar meanings such as job skills and expertise were revised to job expertise based on the KSA (Knowledge, Skill, and Ability), ultimately increasing the sub-indexes from seven to eight. For organizational management capacity, work efficiency, organizational innovativeness, and cooperation of subordinate organizations were eliminated. In light of the opinion that budget management ability and sufficiency of resources have similar meanings and thus must be integrated into a single index, they were revised into resource (budget, personnel, etc.) securement and efficient use. Moreover, new items such as an open culture and strategic human resource management (talent hunt, development, and use) were added, and thus the sub-indexes were ultimately reduced from 17 to 15.

#### Results of Delphi Round 2

Table 2 shows the results of the statistical analysis on individual, middle manager, and organizational PIC. The coefficient of variation of stability was lower than 0.80, indicating that there was a high degree of consensus. The content validity when the scope was set to 4 (slightly valid) and 5 (highly valid) was a minimum of 0.31 for the 14 respondents, indicating that they were all valid. Convergence was lower than 0.50 and close to 0, showing almost no deviation in the collection of opinions, and consensus was higher than 0.70 and close to 1, indicating that all opinions arrived at consensus (Park, 2015). Items that did not satisfy the statistical thresholds were eliminated, and the names of variables were changed or new items were added based on the experts’ opinions (see red-colored items).

**TABLE 2:** Results of Delphi Round 2.

Individual PIC			Secure validity	Content validity	Degree of consensus		
Criteria	Designation	Capacity index	Coefficient of variation	CVR	Quadrant factor	Convergent diagram	Consensual diagram
Individual characteristic capacity	Value innovation	Public value (state view, public service view, ethical belief)	0.08	1	4.75–5	0.12	0.95
		**Self-control ability**	0.29	**0.71**	**4–5**	**0.50**	**0.75**
		Accountability (administrative, legal, professional, moral)	0.12	0.85	4.75–5	0.12	0.95
	**Sum**		**0.10**	**0.71**	**4.25–5**	**0.37**	**0.84**
	Creativity political sense	**Political judgment force**	**0.26**	**0.71**	**4–5**	**0.50**	**0.75**
		Adaptability (job, organization, interpersonal)	0.11	1	4–5	0.50	0.75
		Insight	0.16	0.71	4–5	0.50	0.75
	**Sum**		**0.13**	**0.42**	**3.66–4.66**	**0.50**	**0.79**
	Innovation achievement-oriented	Challenging	0.05	1	5	0	1
		**Enterprising**	**0.09**	**1**	**4–5**	**0.50**	**0.75**
		Goal-oriented	0.07	1	5	0	1
	**Sum**		**0.06**	**1**	**4.66–5**	**0.22**	**0.92**
Job performance	Innovative work performance	Job expertise based on KSA	0.13	0.85	4–5	0.50	0.75
		Public service mindset toward citizens	0.17	0.71	4–5	0.50	0.78
		Display self-leadership to perform duties efficiently	0.16	0.71	4–5	0.50	0.75
		**Foreign language skill to acquire advanced cases**	**0.30**	**0**	**3–4.25**	**0.62**	**0.65**
		Performance-centered duties	0.11	1	4–5	0.50	0.78
		**Policy and business briefing ability**	**0.28**	**0.42**	**3–5**	**1**	**0.50**
	**Sum**		**0.12**	**0.57**	**3.91–4.5**	**0.31**	**0.86**
	Innovation planning	Strategic thinking	0.07	1	5	0	1
		**Policy management ability**	**0.43**	**0.42**	**3.75–5**	**0.62**	**0.69**
		Creative problem-solving ability	0.14	0.85	4–5	0.50	0.75
		Crisis and change management ability	0.12	0.85	4.75–5	0.12	0.95
		**Rational decision making**	**0.14**	**0.85**	**4–5**	**0.50**	**0.78**
	**Sum**		**0.10**	**0.71**	**4–4.8**	**0.40**	**0.83**
	Smart Information Management	Business information and share and tacit knowledge building	0.16	0.71	4–5	0.50	0.75
Relationship formation	Innovation win–win relation-ship formation	Collaborative work performance	0.07	1	5	0	1
		Empathic ability	0.23	0.85	4.75–5	0.12	0.95
		**Network formation ability**	**0.37**	**0.57**	**3.5–5**	**0.75**	**0.75**
		**Conflict management**	**0.19**	**0.85**	**4–5**	**0.50**	**0.78**
		Communication skills	0.24	0.85	4–5	0.50	0.78
	**Sum**		**0.12**	**0.71**	**4.2–4.85**	**0.32**	**0.86**
	Innovative network formation	Stakeholder integration ability	0.28	0.57	3.75–5	0.12	0.73
		**Peddling political influence**	**0.48**	**0.14**	**1–4**	**0.66**	**0**
	**Sum**		**0.30**	**0.14**	**2.87–4.5**	**0.81**	**0.60**
Individual characteristic capacity	Value innovation	Accountability (administrative, legal, and professional, moral)	0.12	0.85	5	0	1
		Distribution and process fairness	0.26	0.71	4–5	0.50	0.80
		Ethics as a public official	0.24	0.85	4–5	0.50	0.80
		Seek social values	0.07	1	5	0	1
		Spirit of sacrifice to the organization and country	0.28	0.71	3.75–5	0.62	0.73
		**Sum**	**0.11**	**0.57**	**4.1–5**	**0.45**	**0.81**
	Innovation achievement-oriented	Goal-oriented	0.08	1	4.75–5	0.12	0.95
		Public entrepreneurship (innovation, initiative, and risk taking)	0.09	1	4–5	0.50	0.90
		**Sum**	**0.08**	**1**	**4.5–5**	**0.25**	**0.90**
	Creativity index	Flexible thinking	0.09	1	4–5	0.50	0.80
		Emotional intelligence	0.23	0.57	3.75–5	0.62	0.73
		Positive psychological capital (resilience, hope, self-efficacy, and optimism)	0.19	0.85	4–5	0.50	0.78
		**Sum**	**0.13**	**0.71**	**4–5**	**0.50**	**0.78**
Job performance	Innovative work performance	Strategy for actioning vision	0.13	0.85	4–5	0.50	0.80
		Environmental analysis ability	0.10	1	4–5	0.50	0.80
		Business convergence skill	0.09	1	4–4.25	0.12	0.94
		Job expertise based on KSA	0.08	1	4.75–5	0.12	0.95
		**Sum**	**0.06**	**1**	**4.37–4.5**	**0.06**	**0.98**
	Innovative planning	Ability to develop and manage policies	0.08	1	4.75–5	0.12	0.95
		Creative problem-solving skills	0.09	1	4–5	0.50	0.80
		Reasonable decision making	0.09	1	4–5	0.50	0.80
		Future forecasting ability	0.13	0.85	4–5	0.50	0.80
		**Sum**	**0.08**	**0.85**	**4.5–5**	**0.25**	**0.90**
	Innovative motivation	Persuasive power for member motivation	0.13	0.85	4–5	0.50	0.80
		Coaching and feedback about subordinates’ work	0.09	1	4–5	0.50	0.80
		**Sum**	**0.10**	**0.85**	**4.37–5**	**0.31**	**0.88**
Org. management	Innovative process	Establishment and propagation of vision	0.10	1	4–5	0.50	0.80
		Organizational learning management	0.14	0.85	4–5	0.50	0.75
		Strategic resource management (training, development, and utilization)	0.13	0.85	4–5	0.50	0.78
		Establish and manage the organization and members’ goals	0.17	0.71	4–5	0.50	0.78
		Smartness of change response	0.19	0.85	4–5	0.50	0.75
		Efficient authority delegation	0.09	1	4–5	0.50	0.80
		**Sum**	**0.09**	**0.85**	**4.16–4.8**	**0.35**	**0.85**
	Innovative conflict management strategy	Manage and mediate conflicts	0.07	1	5	0	1
		Build inclusive teamwork	0.15	0.85	4.75–5	0.12	0.95
		Manage the diverse needs of the members	0.16	0.71	4–5	0.50	0.80
		**Sum**	**0.10**	**0.85**	**4.3–5**	**0.35**	**0.86**
Relationship formation	Innovation win–win relation-ship formation	Participate in official and non-official mentoring	0.29	0.42	3–5	1	0.50
		Mediator of vertical and horizontal communication	0.09	1	4–5	0.50	0.80
		Listening to members’ opinions	0.28	0.57	3.75–5	0.62	0.75
		Builds social capital in the org. (trust and network)	0.09	1	4–5	0.50	0.80
		**Followership**	**0.26**	**0.57**	**4–5**	**0.50**	**0.78**
		**Sum**	**0.09**	**0.71**	**4.15–4.6**	**0.25**	**0.89**

**Organizational PIC**			**Secure validity**	**Content validity**	**Degree of consensus**		
**Criteria**	**Designation**	**Capacity index**	**Coefficient of variation**	**CVR**	**Quadrant factor**	**Convergent diagram**	**Consensual diagram**

Org. goal	Establish innovation model	Establish and share vision	0.53	1	4.75–5	0.12	0.95
		Establish and implement strategies	0.53	1	4.75–5	0.12	0.95
		**Sum**	**0.53**	**1**	**4.75–5**	**0.12**	**0.95**
	Innovative support	Develop and use core competencies	0.10	1	4–5	0.50	0.80
		Actively support efficient policy implementation	0.53	1	4.75–5	0.12	0.95
		**Develop policies to achieve organizational goals**	**0.14**	**0.85**	**4–5**	**0.50**	**0.80**
		**Sum**	**0.09**	**0.85**	**4.5–5**	**0.25**	**0.90**
Org. members Individual characteristic capacity andJob performance	Value innovation	Willingness of social value realization	0.08	1	4.75–5	0.12	0.95
		Owner spirit	0.24	0.85	4–5	0.50	0.80
		**Sum**	**0.07**	**1**	**4.5–5**	**0.25**	**0.90**
	Innovation achievement-oriented	Public service mindset toward the citizens	0.08	1	4.75–5	0.12	0.95
		Display self-leadership for job duty	0.11	1	4–5	0.50	0.80
		Performance-oriented followership/leadership	0.13	0.85	4–5	0.50	0.80
		Ability to manage public service quality	0.14	0.85	4–5	0.50	0.80
		Job expertise based on KSA	0.13	0.85	4–5	0.50	0.80
		Actively change management in response to environment change	0.08	1	4.75–5	0.12	0.95
		**Sum**	**0.09**	**0.57**	**4.29–5**	**0.35**	**0.85**
Org. management	Innovative process	Intelligent public management	0.26	0.71	4–5	0.50	0.78
		Enabling learning organization	0.11	1	4–5	0.50	0.80
		Simplification of decision process	0.16	0.71	4–5	0.50	0.80
		Strategic resource management (training, development, and utilization)	0.07	1	5	0	1
		Security and efficient utilization of resources (budget, manpower, etc.)	0.41	0.57	3.25–5	0.87	0.65
		Active vertical and horizontal communication	0.08	1	4.75–5	0.12	0.95
		**Sum**	**0.07**	**0.85**	**4.29–4.7**	**0.20**	**0.91**
	Innovative culture	Cooperation-oriented culture for organizational social capital construction	0	1	5	0	1
		Future-oriented culture for dynamic organization building	0.08	1	4.75–5	0.12	0.95
		Flexible organizational structure for rigid culture mitigation	0.45	0.42	1.75–5	1.6	0.35
		Open culture for information acquisition and innovation creation	0.12	0.85	4.75–5	0.12	0.95
		Support a flexible work environment	0.19	0.85	4–5	0.50	0.80
						1	
					3–5		0.50
				0.42			
			0.24				
		Convergence culture through balanced personnel management					
						0.50	
					4–5		0.80
				0.71			
			0.16				
		Performance-oriented culture for improving public service quality					
						**0.37**	
					**4.1–4.85**		**0.84**
				**0.85**			
			**0.10**				
		**Sum**					
	Innovation network formation	Political support/tact for securing resources	0.28	0.57	3.75–5	0.62	0.69
		Internal and external cooperative network management	0.10	1	4–5	0.50	0.80
		**Sum**	**0.15**	**0.57**	**3.87–5**	**0.56**	**0.75**

*Bold: Significant.*

## Study 2: Validating Public Innovation Capacity

### Research Method

#### Participants

Based on the Delphi results, the study developed a survey questionnaire on PIC (see Supplementary Appendix). The questionnaire was constructed in sentence form to better clarify the three dimensions of PIC. A total of 1,290 questionnaires were distributed to 43 central government agencies; 477 were collected from 30 agencies and used for the study. The distributions of the socio-demographic characteristics of the sample are provided in Table 3. Male comprised 69.81% of the sample and female comprised 29.35%. More people were in their 30s (26.00%) than in any other age group. In terms of education, “bachelor” was the most common with 67.92%, followed by “master” (20.55%). In terms of the respondents’ rank, “grade 6” was the most frequent (52.62%), followed by “grade 5” (20.34%) and “grade 7” (15.51%). In terms of job tenure, “over 15 years” was the most common, with 46.96%.

**TABLE 3:** Characteristics of the survey participants.

Variables	Classify	Frequency (%)	Variables	Classification	Frequency (%)
Gender	Male	333 (69.81)	Education	Less than college	34 (7.13)
	Female	140 (29.35)			
				Bachelor	324 (67.92)
	No response	4 (0.84)			
Age	20s	30 (6.29)		Master	98 (20.55)
	30s	124 (26.00)			
				Doctor	17 (3.56)
	40s	236 (49.48)			
	50s	95 (19.92)			
				No response	4 (0.83)
	No response	2 (0.42)			
Job tenure	Under 3 years	49 (10.27)	Rank	Grade 9 (Assistant)	6 (1.26)
				Grade 8 (Senior Assistant)	17 (3.56)
	3–5 years	30 (6.29)			
				Grade 7 (Manager)	74 (15.51)
	5–10 years	68 (14.26)			
				Grade 6 (Senior Manager)	251 (52.62)
				Grade 5 (Deputy Director)	97 (20.34)
	10–15 years	104 (21.80)			
				Grade 4 (Senior Deputy Director)	22 (4.61)
	Over 15 years	224 (46.96)			
				Over Grade 3 (Senior Civil Service)	4 (0.84)
	No response	2 (0.42)			
				No response	6 (1.26)
***N*: 477**					

#### Exploratory Factor Analysis and Confirmatory Factor Analysis

We employed an exploratory factor analysis (EFA) and confirmatory factor analysis (CFA) to operationalize the PIC variables using public employees’ survey data that was collected from July to October 2018. First, the study conducted an EFA using SPSS 21.0 and confirmed the reliability of the research variables (i.e., internal consistency) through the eigenvalues, factor loadings, % of variation, and Cronbach’s alpha. Then, in order to confirm the validity (i.e., external consistency), the study conducted a CFA using AMOS 21.0 and checked the goodness of fit based on the suggested cut-off value. In addition, the study confirmed the convergent validity results using average variance extracted (AVE; over 0.05) and critical reliability (CR; over 0.70).

### Results of the Reliability and Validity Tests

The Cronbach’s alpha of all research variables was higher than 0.70, the AVE was higher than 0.50, and the construct reliability was higher than 0.70, thereby ensuring the reliability and convergent validity of the research variables. The AVE of organizational support capacity was 0.445, which was quite a lot lower than the threshold, but it was still used as a research variable because the Cronbach’s alpha and construct validity were satisfactory (see Table 4).

**TABLE 4:** Results of the reliability and validity tests.

Classification	PIC			Cronbach’s alpha		AVE		CR
Individual	Value innovation capacity			0.843		0.694		0.910
	Job innovation capacity			0.924		0.540		0.837
	Relations innovation capacity			0.895		0.674		0.899
Middle manager	Job innovation capacity			0.945		0.566		0.857
	Organizational management innovation capacity			0.944		0.612		0.876
Organization	Organizational goal innovation capacity			0.937		0.690		0.910
	Human resource innovation capacity			0.910		0.620		0.885
	Organizational support innovation capacity			0.940		0.445		0.793
**Results of the CFA**								
Suggested cut-off value	df	*x* ^2^	*x*^2^/df	NFI	TLI	CFI	IFI	RMSEA
	–	–	<3	>0.90	>0.90	>0.90	>0.90	<0.08
Individual	98	289.16	3.97	0.936	0.940	0.951	0.951	0.077
Middle manager	226	847.49	3.75	0.927	0.939	0.946	0.946	0.074
Organization	165	666.57	4.04	0.925	0.934	0.942	0.943	0.078

To determine the factor structure of PIC at the individual, middle manager, and organizational levels and verify the construct validity, the study conducted a higher−order CFA using AMOS 21.0. This was to verify the theoretical validity and legitimacy of PIC deducted through the EFA. To verify the results of the CFA, the study verified the validity of the research variables based on goodness of fit such as absolute, relative, and simplicity (Lee et al., 2019). *x*^2^, NFI, TLI, CFI, IFI, and RMSEA were identified to verify the goodness of fit of the research variables in PIC. The results of the CFA satisfied the acceptable fit index presented, thereby ensuring the convergent validity of the sub-variables of PIC at the individual, middle manager, and organizational levels (see Table 4).

#### Discussion

Value innovation capacity in individual PIC is comprised of the fundamental values of public officials such as the value of public office, self-management skills, responsibility, etc.; job innovation capacity is comprised of the behavioral capabilities to perform innovation duties such as insight, taking on challenges, being goal oriented, having an enterprising spirit, tenacity, strategic thinking, creative problem-solving skills, analytical decision making, etc.; and relations innovation capacity is comprised of the capabilities to manage a personal network within an organization such as performing collaborative duties, empathic ability, conflict management, communication skills, etc.

Job innovation capacity in middle manager PIC is comprised of the behavioral capabilities of middle managers that can innovatively perform their duties based on ethical knowledge and expertise in responsibility, ethics, and meeting the public interest; being goal oriented; showing public entrepreneurship; having analytical skills, job expertise, policy development skills, creative problem-solving skills, etc. Organizational management innovation capacity is comprised of the capabilities to strategically manage the organization considering the diverse needs of members, such as the ability to predict the future and provide a vision; practicing organizational learning management and strategic human resource management; distributing authority, managing conflicts, etc.

Organizational goal innovation capacity is comprised of organizational PIC that contributes to organizational innovation based on the establishment of strategic management models, such as establishing a vision and strategies for government departments, developing and using core competencies, supporting efficient policy implementation, developing policies, etc. Human resource innovation capacity is comprised of the behavioral capabilities of organizational members who innovatively perform their duties based on ethical knowledge and expertise, such as the creation of public interest, ownership, displays of self-leadership, a public service mindset, performance-oriented followership, public service quality management skills, etc. Finally, organizational management innovation capacity is comprised of innovative organizational cultural capacities that support organizational members in bringing about innovation, such as data-based decision making, strategic human resource management, flexible work environment support, active communication, Holacracy, openness, a convergence culture, systematic network management, etc.

## Conclusion

Previous studies have diversely topologized innovation capacity such as internal/external resources (Romijn and Albaladejo, 2002), structural/functional capacity, internal management/external innovation capacity, and internal innovation/external innovation capacity. This study limited the research scope to public organizations and topologized PIC by classifying it into three levels: individual, middle manager, and organizational. According to Jing and Osborne (2017), innovation in the public sector enables service providers to provide higher-quality services by disclosing public information to the beneficiaries (nations or citizens) and providing more public services. Furthermore, the authors emphasized the role of public officials as “innovators,” claiming that innovators in the public sector are the members of public organizations. In this view, the study decided that elucidating the PIC of individuals, middle managers, and organizations, who are the innovators in the public sector, is the most fundamental stage in discussing government innovation; thus, the study topologized PIC. Sørensen and Torfing (2011) stated that innovation is divided into three phases: first change (producing or delivering good products, services, or solutions), second change (changing the repertoire such as services and organizational customs), and third change (transforming the purpose of policies and theories of programs). PIC in this study could connote all three phases of innovation change. For example, the strategic thinking, creative problem-solving skills, analytical decision making, goal-oriented nature, and public entrepreneurship of individuals and middle managers indicate the first change. Data-based decision making, strategic human resource management, flexible work environment support, active communication, Holacracy, and open and convergence cultures indicate the second change. Establishing a vision and strategies for government departments, developing and using core competencies, supporting efficient policy implementation, and establishing strategic management models such as policies indicate the third change.

Moreover, the sub-factors of individual, middle manager, and organizational PIC connoted the factors of NPM and post-NPM innovation values (Park and Joaquin, 2012). More specifically, public values, ethics, the creation of public interest, empathic ability, communication skills, distribution of authority, conflict management, flexible work environment support, active communication, Holacracy, and open and convergence cultures reflect post-NPM values. Taking on challenges, being goal oriented, having an enterprising spirit, tenacity, strategic thinking, creative problem-solving skills, public entrepreneurship, analytical decision making, performance-oriented followership, and public service quality management skills reflect NPM values.

### Theoretical and Practical Implications

First, this study developed and verified indexes for individual, middle manager, and organizational PIC, and attempted to establish concepts regarding PIC in the public sector. Through typologizing PIC, this study was able to explain the PIC of individuals, middle managers, and organizations in terms of organizational and human resources theories, such as self-efficacy, creativity, intrinsic motivation, job crafting, trait theory, behavioral theory, contingency theory, the behavioral life-cycle model, resource-based theory, etc. The theoretical background at the individual, middle manager, and organizational levels in the sub-factors of PIC was ultimately deduced from the results of the statistical analyses. For example, there were “I have self-management skills”(self-efficacy), “I have creative problem-solving skills” (creativity), “I am always up for challenges” (intrinsic motivation), and “I have the ability to manage change” (job crafting) at the individual level; “my middle manager is goal-oriented” (trait theory), “my middle manager manages and mediates conflicts” (behavioral theory), “my middle manager has strategies to fulfill the vision” (contingency theory), and “my middle manager managers the diverse needs of the members” (behavioral life-cycle model) at the middle manager level; and “our department develops policies to achieve organizational goals” (resource-based theory), “the members of our department have a public service mindset toward the citizens” (resource-based theory), and “our department makes decisions based on data” (resource-based theory) at the organizational level. These results have significance in that they interpreted the organizational and human resources theories in terms of various aspects of the new concept of PIC, thereby expanding the application scope of the theories.

Second, the PIC developed in this study can be used as a diagnostic tool to verify the level of PIC in Korean public organizations. Previous studies presented measurements of the levels in the personnel management system; organizational system; structure or culture, such as recruitment, selection, and evaluation; and diversity management as indexes to measure the level of government or personnel reform (Hong et al., 2008). This study developed indexes of PIC at the individual, middle manager, and organizational levels that can lead and diffuse innovation in the Korean public sector, assuming that the main actors of government innovation are government employees. Moreover, the indexes of PIC presented in this study were developed based on a theoretical review of innovation studies conducted in the public and private sectors, thereby including the universality of the public sector (fulfillment of the public interest, expansion of public values, etc.) and specificity of the private sector (creativity, entrepreneurship, etc.), which can be applied to various organizations. Thus, indexes of PIC can also be indexes used to measure the PIC of public enterprises, lower-level local governments, and even social enterprises and central government agencies.

Finally, in order to enhance innovation capabilities at the individual, middle manager and organizational level, it is necessary to more actively implement the strategic human resource management policy. The linkage among human resource management functions must be secured so that the strategic human resource management functions within the organization can be effectively implemented. In other words, based on the systematic definition of competency required for individual jobs and series, the system of securing human resources – training and development – performance management – compensation should be integrated and operated.

### Research Limitations and Future Research Directions

This study limited its subject to the Korean public sector in typologizing and verifying the PIC. Furthermore, it failed to convey all of the opinions in the public sector, such as those of public enterprises and lower-level local governments, while conducting the survey. In other words, there may be insufficient grounds to generalize the results of this study in the Korean public sector. To elaborate and secure the validity of the PIC indexes in this study, future research must distribute the questionnaire to all public organizations and restructure the sub-indexes. Moreover, as discussed in the theoretical background section, there are various types of public service and methods of providing them, and thus discussing PIC only within public organizations may have had a negative impact on expanding the scope of research. Therefore, future research must diversify the PIC indexes according to the various types of public service (everyday service, protective service, developmental service, minimum social security service, etc.), and develop indexes based on the actors that provide public services (public sector, private sector, NGOs, social enterprises, etc.). It is necessary to develop indexes of PIC that coincide with the characteristics of agencies by expanding the scope of research to public enterprises, lower-level local governments, executive agencies, public research institutes, social enterprises, and NGOs that require innovation.

## Data Availability Statement

The raw data supporting the conclusions of this article will be made available by the authors, without undue reservation.

## Ethics Statement

Ethical review and approval was not required for the study on human participants in accordance with the local legislation and institutional requirements. Written informed consent for participation was not required for this study in accordance with the national legislation and the institutional requirements.

## Author Contributions

Both authors listed have made a substantial, direct, and intellectual contribution to the work, and approved it for publication.

## Conflict of Interest

The authors declare that the research was conducted in the absence of any commercial or financial relationships that could be construed as a potential conflict of interest.

## Publisher’s Note

All claims expressed in this article are solely those of the authors and do not necessarily represent those of their affiliated organizations, or those of the publisher, the editors and the reviewers. Any product that may be evaluated in this article, or claim that may be made by its manufacturer, is not guaranteed or endorsed by the publisher.

## Footnotes

1 The validity of Delphi studies is dependent on the competence and knowledge of panel members; therefore, a heterogeneous group of experts who possess a wide understanding of the desired subject matter should be selected (Lamm et al., 2021, p. 3). Primarily, with the use of the Delphi technique, the issues generated are limited to the insights of the expert panel members (Lamm et al., 2021, p. 10). Although measures were taken to reduce bias and assemble a heterogenous panel, the results may not be generalizable to all contexts (Lamm et al., 2021, p. 10).

2 Expert selection: identified relevant public official and academic experts, related to work and research on PIC.

3 The items in the first-round Delphi survey consisted of 34 items for individual PIC, 32 items for middle manager PIC, and 29 items for organization PIC deduced based on word appearance frequency on content analysis. The items extracted a variable with a high frequency (appearing over three times), using abstracts (all words used in a sentence [average of 10 sentences]) via network analysis. Individual, middle manager, and organizational PIC were classified based on the classification standard of the capacity groups examined in the theoretical background section: individual PIC was classified into individual attribute capacity, job performance capacity, organizational management capacity, and interpersonal capacity; middle manager PIC into individual attribute capacity, job performance capacity, organizational management capacity, and interpersonal capacity; and organizational PIC into organizational goal capacity, job performance capacity, and organizational management capacity.

4 Based on the questionnaire collected in the second-round Delphi survey, the basic statistics (mean, standard deviation, and median) were analyzed using SPSS 21.0. we also conducted a content validity analysis to ensure the validity of the survey items; performed a stability analysis to determine whether to repeat the Delphi survey; and calculated the quartile, convergence, and consensus to analyze the degree of consensus among the experts.
