# Supplementary material for: Deriving Public Innovation Capacity: Evidence From the Korean Public Sector
Source: Front Psychol. 2022 Jun 2;13:898399. doi: 10.3389/fpsyg.2022.898399 (PMC9201325; doi:10.3389/fpsyg.2022.898399)
Supplement: Supplementary file 1 [file Table_1.DOCX]

**Appendix. Survey Questionnaire**

| **Level** | **Variable** | **Contents** |
| --- | --- | --- |
| Individual | Individual  Characteristic | 1. I cultivate public values (state view, public service view, ethical belief). |
|  |  | 2. I have self-management skills. |
|  |  | 3. I have a sense of responsibility (administrative, legal, professional, moral). |
|  |  | 4. I have political judgment. |
|  |  | 5. I have adaptability (job, organization, interpersonal). |
|  |  | 6. I have insight. |
|  |  | 7. I am always up for challenges. |
|  |  | 8. I am enterprising. |
|  |  | 9. I am goal oriented. |
|  |  | 10. I have tenacity. |
|  | Job  Characteristic | 1. I have job expertise. |
|  |  | 2. I have a public service mindset toward citizens. |
|  |  | 3. I display self-leadership to perform duties efficiently. |
|  |  | 4. I share (use) work information. |
|  |  | 5. I carry out performance-centered duties. |
|  |  | 6. I have the ability to manage change. |
|  |  | 7. I handle administrative affairs actively. |
|  |  | 8. I think strategically. |
|  |  | 9. I have creative problem-solving skills. |
|  |  | 10. I make decisions analytically. |
|  | Relations  Characteristic | 1. I perform duties collaboratively. |
|  |  | 2. I have the ability to empathize with others. |
|  |  | 3. I have the ability to mediate conflicts. |
|  |  | 4. I have communication skills. |
|  |  | 5. I have the ability to communicate with stakeholders. |
|  |  | 6. I exert influence within our organization. |
| Middle  Manager  Leadership | Individual  Characteristic | 1. My middle manager has a sense of responsibility (administrative, legal, professional, moral). |
|  |  | 2. My middle manager has ethics as a public official. |
|  |  | 3. My middle manager seeks social values to meet the public interest. |
|  |  | 4. My middle manager is goal oriented. |
|  |  | 5. My middle manager has public entrepreneurship skills (innovation, initiative, risk taking). |
|  |  | 6. My middle manager thinks flexibly. |
|  |  | 7. My middle manager has positive psychological capital (resilience, hope, self-efficacy, optimism). |
|  | Job  Characteristic | 1. My middle manager has the ability to analyze the environment. |
|  |  | 2. My middle manager is fair. |
|  |  | 3. My middle manager has business convergence skills. |
|  |  | 4. My middle manager has job expertise. |
|  |  | 5. My middle manager handles administrative affairs actively. |
|  |  | 6. My middle manager has the ability to develop and manage policies. |
|  |  | 7. My middle manager has creative problem-solving skills. |
|  |  | 8. My middle manager makes reasonable decisions. |
|  |  | 9. My middle manager has the ability to predict the future in the quaternary industries. |
|  |  | 10. My middle manager motivates the members. |
|  |  | 11. My middle manager provides coaching and feedback. |
|  | Org.  Management  Characteristic | 1. My middle manager provides a vision. |
|  |  | 2. My middle manager has strategies to fulfill the vision. |
|  |  | 3. My middle manager manages organizational learning. |
|  |  | 4. My middle manager strategically manages human resources (talent hunt, development, promotion). |
|  |  | 5. My middle manager builds an innovative organizational culture. |
|  |  | 6. My middle manager is responsive to change. |
|  |  | 7. My middle manager distributes authority efficiently. |
|  | Relations  Characteristic | 1. My middle manager manages and mediates conflicts. |
|  |  | 2. My middle manager builds inclusive teamwork. |
|  |  | 3. My middle manager manages the diverse needs of the members. |
|  |  | 4. My middle manager is a mediator of vertical and horizontal communication. |
|  |  | 5. My middle manager builds trust. |
|  |  | 6. My middle manager induces followership. |
| Organization | Org.  Goal | 1. Our department establishes and shares a vision. |
|  |  | 2. Our department establishes and implements strategies. |
|  |  | 3. Our department develops and uses core competencies. |
|  |  | 4. Our department actively supports efficient policy implementation. |
|  |  | 5. Our department develops policies to achieve organizational goals. |
|  | HR  Competency | 1. The members of our department have a public service mindset toward the citizens. |
|  |  | 2. The members of our department have ownership. |
|  |  | 3. The members of our department display self-leadership. |
|  |  | 4. The members of our department fulfills social values by creating public interest. |
|  |  | 5. The members of our department display performance-oriented followership/leadership. |
|  |  | 6. The members of our department have the ability to manage public service quality. |
|  |  | 7. The members of our department have job expertise. |
|  |  | 8. The members of our department deal with environmental change. |
|  | Org.  Management | 1. Our department has the administrative tasks computerized. |
|  |  | 2. Our department has the learning organization activated. |
|  |  | 3. Our department makes decisions based on data. |
|  |  | 4. Our department strategically manages human resources (talent hunt, development, promotion, motivation). |
|  |  | 5. Our department supports a flexible work environment. |
|  |  | 6. Our department has active vertical and horizontal communication. |
|  |  | 7. Our department has a collaboration-oriented culture. |
|  |  | 8. Our department has a future-oriented Holacracy* culture. |
|  |  | 9. Our department has an open culture. |
|  |  | 10. Our department has a performance-oriented culture. |
|  |  | 11. Our department has a convergence culture featuring balanced personnel management. |
|  |  | 12. Our department systematically manages the collaborative network among organizations. |
|  |  | 13. Our department exerts political influence. |

*Middle managers are perceived relatively depending on the respondent’s job title or position

(e.g., A section chief perceives the general manager as the middle manager/an action officer perceives a deputy director or section chief as the middle manager)

*Holacracy: A form of organization in which the decision-making process is distributed throughout the entire organization
